# Barriers to Community-Based Primary Health Care Delivery in Urban China: A Systematic Mapping Review

**DOI:** 10.3390/ijerph191912701

**Published:** 2022-10-04

**Authors:** Bo Li, Juan Chen

**Affiliations:** 1 Department of Applied Social Sciences, The Hong Kong Polytechnic University, Hong Kong, China; 2 Mental Health Research Centre, The Hong Kong Polytechnic University, Hong Kong, China

**Keywords:** primary health care, care delivery, community health, systematic mapping review, urban China

## Abstract

Providing access to a range of basic health services, community-based primary health care (CB-PHC) plays a vital role in achieving the goal of health for all. Driven by a strong political commitment, China’s CB-PHC progress in the past decade has been swift and impressive. However, a well-functioning delivery system for care has yet to be established. This systematic mapping review synthesizes selected evidence on barriers to CB-PHC delivery in urban China and draws lessons for policy development. We performed searches on five electronic databases: CINAHL, MEDLINE, Scopus, Web of Science, and China National Knowledge Infrastructure, and included studies published between 2012 and 2021. The Downs and Black and Critical Appraisal Skills Program checklists were used to assess the quality of eligible papers. We conducted our searches and syntheses following the framework set out in the Primary Health Care Performance Initiative (PHCPI). We synthesized the results of the included studies using a thematic narrative approach and reported according to PRISMA guidelines. Six salient barriers arose from our syntheses of 67 papers: lack of comprehensive health insurance schemes, lack of public awareness, superficial care relationships, gaps in communication, staff shortages and poor training, and second-rate equipment. These barriers are grouped into three subdomains following the PHCPI framework: access, people-centered care, and organization and management. A host of negative impacts of these barriers on community-based health care were also identified. It was not possible to determine clear causes of these barriers from the contributing evidence because of the lack of conceptual frameworks and research methods constraints. Non-eastern regions of China and access-related barriers require further exploration. It follows that, at the national level, the problems are likely more severe than the research suggests.

## 1. Introduction

**Figure 1:**
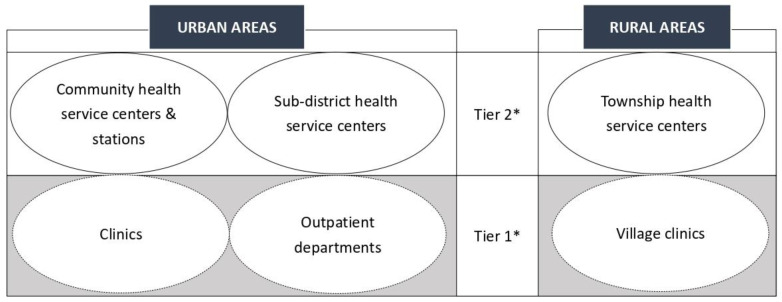
Primary health care providers in China. * China runs a three-tier healthcare system, and hospitals are at the top (i.e., tier 3).

As a whole-of-society approach, primary health care (PHC) addresses the majority of a person’s health needs throughout their lifetime, including health promotion, disease prevention, treatment, rehabilitation, and palliative care [1]. In 1978, the Alma-Ata Declaration committed to global PHC promotion [2]. Forty years later, the 2018 Astana Declaration reconfirmed the centrality of PHC in safeguarding human health [3]. With its inclusive approach, PHC is viewed as an essential reform to healthcare systems that will lead to high-quality universal health coverage [4]. Following England, which pioneered PHC in the 1920 Dawson Report, other wealthy countries have made notable progress in establishing PHC systems. However, many low- and middle-income countries lag behind, primarily hampered by a lack of human and financial resources [3]. As the world’s most populous country with a rapidly expanding global influence, China’s success in providing PHC services is vital not only to the health of its population but also to that of newly-industrialized economies that wish to emulate China’s PHC pattern [5]. Figure 1 shows PHC providers in China.

The comprehensive market-oriented reforms, initiated in the late 1970s, have led to internal migration and rapid urbanization in China on a scale unparalleled in human history [6]. At the outset of the reforms, only 18% of China’s population dwelt in urban areas; in the past four decades, China’s urban population has skyrocketed and, currently, exceeds 900 million, representing over 60% of its entire population [7]. The rapid growth in the urban population has exerted soaring pressure on healthcare infrastructure in urban China.

The privatized reforms in the healthcare sector, implemented between the mid-1980s and the late 1990s, further undermined China’s healthcare system [8]. Such privatization, leading to health impoverishment and widening health inequities, has been subject to extensive criticism. As a result of this, the Chinese government started reorganizing the country’s PHC system in 1997 by, notably, developing community-based PHC (CB-PHC) in urban areas [9]. Consequently, the providers of CB-PHC—community health centers and stations—have proliferated in cities and towns. Table 1 shows key services provided by CB-PHC and non-CB-PHC organizations in urban China, suggesting the extensiveness of services in CB-PHC settings.

The latest round of comprehensive health reforms, launched in 2009, has further accelerated the development of CB-PHC in China [10]. Certainly, the Chinese government has demonstrated its commitment to creating equitable, affordable, accessible, and high-quality CB-PHC services by introducing various policy directives and proactive public programs [11,12]. Nevertheless, despite the vast resources invested and the ensuing commendable progress, China has not yet developed a competent CB-PHC system comparable to its Western counterparts such as the United States, United Kingdom, and Netherlands [13,14]. Unnecessary injections, irrational diagnoses, and inexperienced practitioners continue to characterize China’s CB-PHC organizations. Further, complex neighborhood relationships and cryptic acquaintance networks within the communities undermine the standardization and professionalization of CB-PHC, ultimately impairing the quality of the service. Consequently, the trust of residents in CB-PHC remains low, and a trust-based care relationship is still lacking in CB-PHC settings. As an essential segment of the Chinese public health system, CB-PHC plays the role of “gatekeeper” for Chinese citizens’ health. As the starting point for healthcare, it is vital for promoting the population’s health, and its effectiveness and efficiency are critical determinants of treatment outcomes at all levels of care. In China’s hierarchical public health system, CB-PHC is also responsible for monitoring, collecting, and reporting local public health information under the guidance of higher-level medical institutions and government sectors. So, a well-functioning CB-PHC system is necessary for establishing an effective public health system in urban China.

To date, the reviews of China’s CB-PHC have focused primarily on quality issues [10,15,16,17], while the barriers to delivery have been poorly synthesized. To present a comprehensive picture and to serve as a reference for future research and policy development, this review synthesizes evidence from the literature on CB-PHC delivery in urban China, which is guided by two research questions: from the perspective of service providers and users, what are the barriers to CB-PHC delivery, and what are the impacts of the barriers on community-based health care? The two research questions were formulated using the SPICE model [18], which was adapted to this review as below: S/setting—urban China, P/perspective—CB-PHC service providers and users, I/phenomenon of interest—barriers to CB-PHC delivery, C/comparison—not applicable, and E/evaluation—impacts of the barriers on community-based health care. Compared to other frameworks often employed to identify review questions (e.g., PICO, CIMO) in medicine and social sciences, the SPICE model is more suitable for our review because it comprises two research characteristics—”setting” (missing in PICO) and “perspective” (missing in CIMO)—which are essential for our searching and syntheses. The SPICE model, indeed, allowed us to frame the review questions more specifically [19].

## 2. Materials and Methods

We synthesized the selected evidence by systematic mapping. Systematic mapping is an ideal method to collate, describe, and catalog existing knowledge [20]. It is particularly useful for establishing a classification scheme, structure clusters of interests, and mapping research trends [21]. Through a systematic mapping review, research gaps under a specific topic can be identified, as well as the consequent need for empirical studies or further reviews [22].

### 2.1. Framework of Searches and Syntheses

**Figure 2:**
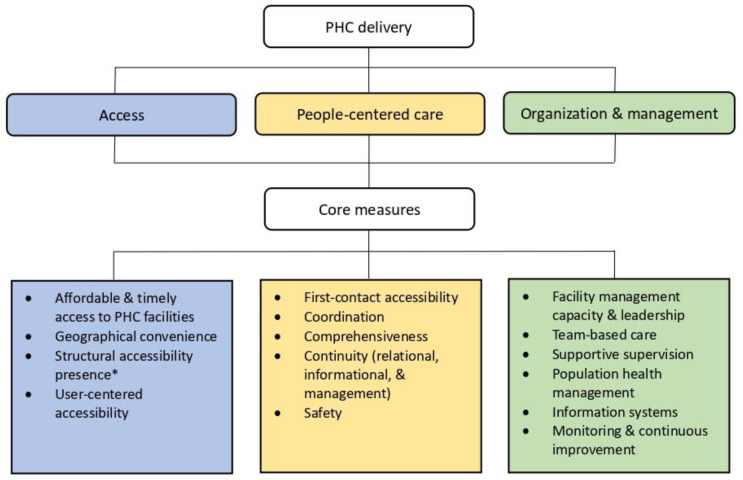
Framework of searches and syntheses, compiled by the authors from the literature [24]. * “Structural accessibility presence” is a dimension used to examine accessibility issues, with a primary focus on the availability of structural elements such as facilities and human resources (e.g., Is there a facility with a provider available for care when it is needed by the community?) [24]. Abbreviation: PHC—primary health care.

We used the framework set out in the Primary Health Care Performance Initiative (PHCPI) to guide our searches and syntheses. The PHCPI framework was developed in collaboration between the Bill and Melinda Gates Foundation, United Nations Children’s Fund, World Bank Group, World Health Organization, and Ariadne Labs to catalyze highly-functioning PHC systems in low- and middle-income countries [23]. The PHCPI framework pinpoints four subdomains for assessment of the performance of PHC delivery: access, availability of effective care, people-centered care, and organization and management [23]. Given the number of existing reviews of the quality of China’s CB-PHC [10,16,17], we did not include the subdomain—availability of effective care—in this review for its primary focus on issues of care quality and effectiveness [24]; instead, we concentrated on the other three subdomains and their core measures for our evidence selection and syntheses (see Figure 2).

### 2.2. Search Strategy

Following the PRISMA 2020 checklist [25], we searched the relevant literature on English and Chinese electronic databases, including CINAHL, MEDLINE, Scopus, Web of Science, and China National Knowledge Infrastructure (CNKI), between March and June 2021. We also performed hand searches of references cited in other reviews [11,15,17] so no relevant studies were omitted. Our searches ranged over the following scholarship domains: health services, health policy, public health, environmental health, and social work. The databases we consulted supported the need for evidence collection on this highly multidisciplinary subject. Given the fact that China’s 2009 health reforms accelerated the development of the country’s CB-PHC at an unprecedented rate, we focused our attention on studies published between 2012 and 2021 (we excluded 2010 and 2011 because we presumed little solid evidence would have been published after just two years following the instruction of the reforms; after all, it takes time to observe policy effects). This time frame of publication, comprising the most recent literature, was inclusive of evidence syntheses and up-to-date research and policy implications. Following the core measures for delivery performance identified in the PHCPI framework, we designed our search strategy and conducted iterative searches of the databases. The terms and queries used during our searches of English databases were refined using a pilot search with CINAHL. We used Boolean operators and Medical Subject Headings to instruct our searches and optimize the breadth of search results. Our key terms and queries can be found in the Supplementary Materials.

### 2.3. Inclusion and Exclusion Criteria

Our searches were limited to peer-reviewed qualitative and quantitative studies published in English and Chinese academic journals between 2012 and 2021. With reference to the SPICE criteria, we excluded studies that did not focus on urban China and/or on the target populations (i.e., CB-PHC service providers and users). To ensure the trustworthiness, relevance, and completeness of evidence, we also excluded the following: (1) studies using non-empirical data; (2) studies using data collected before or in 2009 (the year in which the latest round of comprehensive health reforms was introduced); (3) grey literature (e.g., policy documents, consultancy reports, research notes, commentaries, editorials, letters, correspondence); (4) review articles; (5) pilot studies; and (6) study protocols. Further, with primary attention paid to clinical practices, randomized trials were excluded for their weak pertinence to our review interests.

### 2.4. Screening and Selection

**Figure 3:**
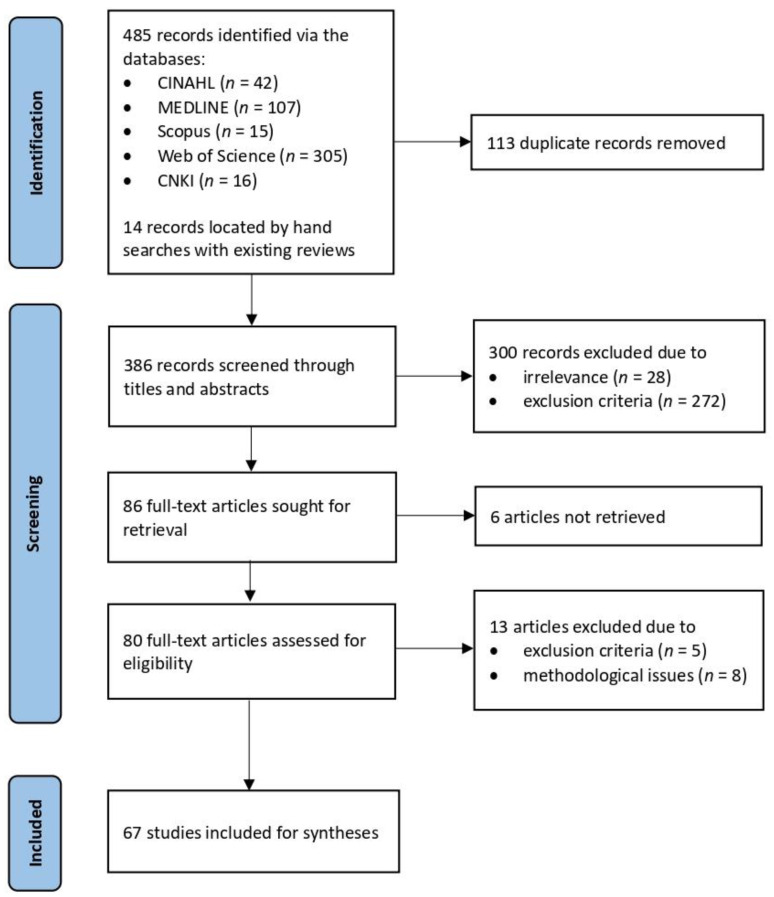
PRISMA flow chart of the study selection process.

A total of 499 articles were identified through our electronic searches of the databases and complementary hand searches. After removing duplicates, the titles and abstracts of the remaining 386 articles were screened by the authors independently. During the first round of screening, 300 articles were excluded based on irrelevance or failure to conform to our criteria. The second round of screening of the main text of the remaining 86 articles was conducted by the authors separately. During this stage, we first removed six articles because the full text of which was not available; we then eliminated five articles based on our exclusion criteria and eight articles which did not meet the methodological standards we established based on the Downs and Black checklist for quantitative studies [26] and the Critical Appraisal Skills Program (CASP) checklist for qualitative studies [27]. Specifically, studies that were judged to have methodological problems in reporting, external validity, or internal validity (bias/confounding), and those that were rated with a total score ≤14 (poor) were excluded [28]. Although a number of quality appraisal tools have been developed, the two checklists we used have been successfully adopted in various studies thanks to their high internal consistency, external validity, and inter-rater reliability (the Downs-Black) [29,30] and succinctness, comprehensiveness, and easier implementation (CASP) [31,32]. These two checklists allowed us to perform a thorough and systematic assessment of studies. The methodological eligibility of studies was assessed by the authors individually, and any disagreement during the selection process was resolved in discussions between the authors. The results of our assessments of the included studies can be retrieved from the Supplementary Materials. This review yielded 67 studies in the final sample. Figure 3 depicts our study selection process.

### 2.5. Data Extraction and Syntheses

We created a library group with EndNote X9 to archive the selected studies. We entered and administered our data using a predetermined data extraction form, by which the aim, sample, setting, and method of each study were initially described. We then summarized the entire body of each study’s resulting section and aggregated them using the form. For qualitative studies, we summarized not only the quotes from the participants’ experiences but also the authors’ interpretations of the quotes. For quantitative studies, we outlined the main statistical outcomes and summarized the authors’ explanations and discussions.

We performed thematic narrative syntheses of the entire body of the resulting section of each study by focusing on the three subdomains of care delivery and their core measures identified in the PHCPI framework. We retrieved and coded the thematic terms using close coding and open coding. The themes and commonalities were then identified through categorization [33]. Finally, we employed axial coding to link the categorized themes and revisited coded terms, themes, and categories to ensure consistency. ATLAS.ti 9.0 was used to manage our coding and analysis. We developed a priori review protocol based on the PRISMA-P checklist [34], which catalogs the rationale, objective, questions, methods, and other essential items of this review (see Supplementary Materials). No registration was completed because this review did not fit the PROSPERO format.

## 3. Results

### 3.1. Characteristics of the Included Studies

Within the time frame of publication we stipulated, 2019 produced the most publications (*n* = 14), accounting for over 20% of the included studies, followed by 2017 with 11 publications. Most of the included studies (*n* = 54) used quantitative research methods; of these, most used a cross-sectional approach (*n* = 47). The number of studies using a cross-section approach further increases when mixed methods studies are considered (*n* = 51), suggesting the popularity of this research method in the field.

Geographically, more than 70% of the included studies (*n* = 48, excluding the nationwide studies) focused on East China. With the exception of Hebei and Hainan, the studies covered all eastern provinces and municipalities in mainland China. Of these, Guangdong received the most attention, with more than half of the studies (*n* = 25) examining CB-PHC delivery in the province. Of the various Guangdong cities considered, Shenzhen, which has experienced dramatic socioeconomic progress in the past four decades, garnered the most studies (*n* = 15), eclipsing the numbers devoted to its counterparts (i.e., Beijing, Shanghai, and Guangzhou).

Twenty-six included studies have investigated barriers to care delivery when treating a particular condition. Notably, nine of these studies dealt with treatments for hypertension, and six studies for treatments for diabetes. These studies highlight the prevalence of these two chronic diseases, which are considered significant public health hazards for residents in urban China, and the important role played by CB-PHC in helping residents to find compatible services for prevention, treatment, rehabilitation, and health education.

**Figure 4:**
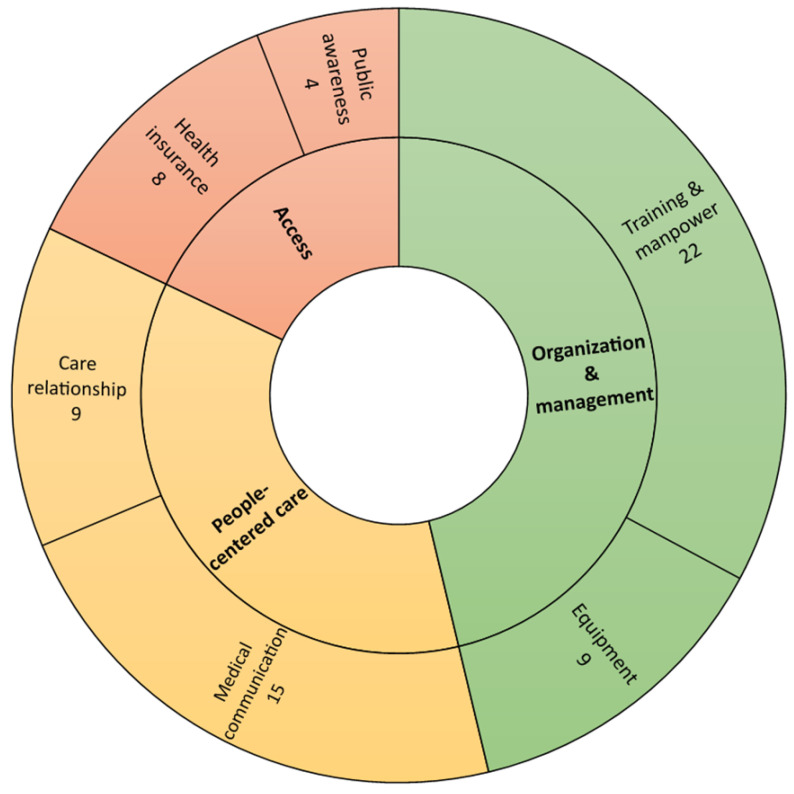
The number of studies focusing on each theme and respective sub-themes.

Following the PHCPI framework, we grouped the included studies according to the themes that arose during our screening process (see Table 2). The grouping reveals that only 12 studies included scrutinized the barriers related to access; most studies focused on the barriers arising from people-centered care (*n* = 24) and organization and management (*n* = 31). Figure 4 visualizes the number of studies focusing on each theme and its sub-theme.

Additionally, half of the studies on barriers arising in people-centered care (*n* = 13) were published between 2012 and 2016. In contrast, those concerned with barriers pertaining to organization and management accounted for most of those published from 2017 to 2021 (*n* = 23, or about 60% of the publications in that period).

Six significant barriers arose from our syntheses of the contributing evidence: lack of comprehensive health insurance schemes, lack of public awareness, superficial care relationships, gaps in communication, staff shortages and poor training, and second-rate equipment. A host of negative impacts of these barriers on community-based health care were identified (see Table 3).

### 3.2. Access

#### 3.2.1. Lack of Comprehensive Health Insurance Schemes

A prominent barrier emerging from our review is the lack of health insurance for all citizens. Access to CB-PHC facilities is contingent on one’s health insurance coverage [35]. The likelihood that people will visit CB-PHC facilities for initial care increases when they are covered by local health insurance. One study shows that over 80% of hypertensive patients with local health insurance received CB-PHC services routinely, compared to 60% without insurance coverage [36]. In addition, uninsured patients are more likely to take medications without supervision, often leading to adverse consequences such as drug interactions and polypharmacy [37]. Those with local health insurance, in contrast, show better outcomes of care and are more likely to adhere to their treatment and take their medications properly [36]. These better outcomes and medication behaviors help to reduce the overuse of hospital services and underuse of CB-PHC [38].

The lack of comprehensive health insurance also prevents some urban residents from obtaining timely access to CB-PHC facilities. Migrants living in urban areas without local household registration (*hukou*), in particular, are vulnerable and largely excluded from the CB-PHC benefits due to the lack of local health insurance [36,37]. Even when migrants are covered by non-local health insurance, that insurance is usually provided in their hometowns where their *hukou* is registered, to where they need to return for reimbursement, often leading to a significant delay in treatment [36]. Such delays contribute to the substandard procedures, outcomes, and perceived quality of care among migrants [36].

One surprising finding of our syntheses is that barriers to access to CB-PHC facilities for non-locals persist even when they are covered by local health insurance. Studies found that, to be reimbursed, migrants with local health insurance must seek services from designated community health organizations before gaining access to higher-tier hospitals, whereas locals are free to shop for services at any level of health care and can receive reimbursement under any circumstances [36]. This stark contrast reveals that health inequities remain pervasive in the urban context, particularly impairing migrants’ access to CB-PHC services [35].

#### 3.2.2. Lack of Public Awareness

Another conspicuous barrier to access is the lack of public awareness regarding CB-PHC. Operating on the assumption that only higher-tier hospitals provide state-of-the-art care, many Chinese people go to large hospitals for their health problems, regardless of the severity of their illnesses [39]. This pattern of health service utilization is difficult to change when residents have little awareness of the nature, benefits, and convenience of CB-PHC.

The lack of public awareness presents a greater obstacle to access among residents with low socioeconomic status (SES), who usually have poorer health literacy and are less able to self-administer care, as a result of the lack of health promotion information dissemination and outreach campaigns in their communities [38,39]. Without these initiatives, residents with low SES do not learn to trust CB-PHC, thereby reinforcing avoidance of its services and overuse of higher-tier hospitals [40].

### 3.3. People-Centered Care

#### 3.3.1. Superficial Care Relationships

A significant barrier identified during our syntheses was the intermittent and superficial care relationships between care providers and users. A germane, dynamic, and sustained care relationship between the two parties has yet to be established [41]. Not surprisingly, this superficiality undermines the generalized trust users have in providers [42]. When there is little trust, there is little effective communication between the two parties during the treatment process, the reasons and methods of treatment become opaque, and users are more likely to suspect the quality of CB-PHC [43].

A superficial care relationship does not lead to a sense of reciprocity and rapport between care providers and users [41,42]. When care providers lose trust in their patients’ cooperation and ability to self-administer medication, they are less likely to respond to those patients’ concerns [39], which, in turn, lowers patients’ trust in CB-PHC, creating a vicious cycle of distrust. As a significant component of social capital, trust is a core dimension of quality of care [44]. Thus, the lack of a trust-based care relationship is very likely to devalue CB-PHC, especially in continuing care.

#### 3.3.2. Gaps in Communication

Another barrier related to people-centered care arises from communication gaps. The mechanisms for archiving medical information in CB-PHC facilities and sharing such information between different layers of healthcare institutions are inadequate and unreliable. Studies have shown that patients often complain about the ways their health records have been used by CB-PHC providers during the process of continuing care [45,46]. Without an effective platform to store and share medical information, timely feedback is not possible [41], treatment becomes inefficient [47], and regular follow-ups are often neglected [41].

In addition, gaps in communication at basic levels of care can result in a lack of adequate records when patients are referred to hospitals. The discontinuity of information across different levels of health care not only compromises the referral mechanisms [48] but can also lead to medical errors and complications throughout the course of care and across various sites of treatment [49]. Achieving a full continuum of health services is costly but, without it, patient care cannot be adequately coordinated and integrated, escalating episodic medical costs and leading to higher rates of hospitalization [49].

### 3.4. Organization and Management

#### 3.4.1. Staff Shortages and Poor Training

One prominent barrier pertaining to organization and management lies in human resources: problems arise from major staff shortages, compounded by a lack of adequate training for existing staff. The scarcity of community health practitioners, not surprisingly, imposes a barrier to the effective delivery of CB-PHC in urban China [45]. This scarcity leads to low utilization rates on the part of patients, coupled with overly heavy workloads on the part of staff [50]. Excessive workloads can lead to burnout and, ultimately, compromise service quality, further making users reluctant to avail themselves of CB-PHC [45].

Moreover, the minimal training provided for active health workers in CB-PHC settings creates a significant barrier to care delivery. Opportunities for continuing education are essential if CB-PHC is to attract and retain health professionals. Studies have shown that the lack of continuing professional training is a key contributor to staff turnover in CB-PHC settings and an obstacle hindering the professional development of health practitioners [13,51]. Community health practitioners are often incapable of giving up-to-date health advice, applying evidence-based practices, understanding and implementing emerging state policies and recommendations, and effectively monitoring antibiotic use, cases of depression, psychological problems, and the ethics of relationships with patients because of inadequate training [51,52,53]. Moreover, the shortage of qualified practitioners has obstructed the progress of the digitalization of CB-PHC services and the modernization of the national healthcare system [54].

#### 3.4.2. Second-Rate Equipment

The final barrier to CB-PHC delivery we identified is the poor quality of equipment. For many years, China has relied on its top-ranking public hospitals to deliver health services to its citizens. This hospital-centered delivery model has prompted hospitals to acquire sophisticated medical equipment. The vast superiority of the equipment available in hospitals is another reason why CB-PHC facilities remain less popular.

The second-rate equipment has, of course, had a negative effect on CB-PHC service delivery. On the side of service providers, second-rate equipment makes updating patients’ health records, accessing medical data, making referrals, and sharing information unnecessarily grueling; the innovative care practices recommended by national guidelines are difficult to follow; and state-of-the-art learning materials and protocols are in short supply [45,51,52,54]. From the patients’ point of view, the lack of advanced equipment is discouraging and reinforces the preference for specialist care in hospitals, contributing to the imbalance of service use between different levels of healthcare institutions [55].

## 4. Discussion

In response to the challenges to public health in the community due to increases in chronic diseases, multi-morbidities, emerging epidemics, and accelerating aging, CB-PHC took on the responsibility to provide a wide range of essential services in pursuit of universal health coverage. The landmark health reforms, introduced in 2009, rolled out and expedited the reconfiguration of China’s CB-PHC network. Following the PHCPI framework, our review not only identified six significant barriers to CB-PHC delivery in urban China but also mapped their negative impacts on community-based health care, which makes this review unique within the literature. Our syntheses of the results of the included studies provide a useful basis for future research and policy development.

In its effort to provide universal health coverage, the Chinese government launched the Urban Employees Basic Medical Insurance (UEBMI) in 1998 and the Urban Residents Basic Medical Insurance (URBMI) in 2007. These two insurance schemes have provided health services to the insured free or at a minimal cost, with the UEBMI available to all urban workers and the URBMI for urban residents without UEBMI coverage. Despite the ensuing remarkable progress, the schemes still have systematic problems attributable to their decentralized and incremental approach [8]. Notably, the lack of a comprehensive health insurance scheme, complicated by the *hukou* regime, erratic local conditions, and rigid stipulations on reimbursement, precludes a large number of migrants from gaining access to CB-PHC in a timely manner, leading to demographic inequities in health [38]. Policy alternatives that can take in more government subsidies and equalize risks are needed to promote the equalization and consolidation of health insurance.

Lack of public awareness also acts as a significant barrier to accessing CB-PHC, especially among those with low SES. Constrained by a poor inhabited environment, poor health literacy, and a self-contemptuous mindset, people with low SES rarely engage in health-promoting events organized by their communities [42,44]. Resources should be directed toward improving objective conditions and subjective incentives of this group to overcome their reluctance and make CB-PHC more inclusive.

Our review demonstrates that the superficial relationship between care providers and users also presents an obstacle to service delivery. Contrary to the rapport often established between community health practitioners and patients in Western societies, care relationships in Chinese community health settings are characterized by low levels of trust, especially on the part of service users. This is mainly attributed to the CB-PHC service’s inability to meet patients’ healthcare demands. Moreover, the Chinese culture of social networks and acquaintance relationships makes the interactions between service providers and users rather intricate [41], which, to some extent, hampers the establishment of a trust-based care relationship. As discussed, trust is a key driver for improving treatment outcomes, so fostering a trust-based care relationship is important in CB-PHC settings. This requires more comprehensive efforts and targeted policy interventions. The current family doctor system would be the cornerstone for establishing a trust-based care relationship if it can be further promoted more systematically.

Good channels of communication are necessary to maintain the quality of continuing care [42]. Timely feedback on medication, scheduling of high-quality treatments, and regular follow-up examinations all depend on informational continuity. In response to the National Basic Public Health Service program, community health organizations established resident health archives for both locals and migrants who have resided in the communities for over six months, setting up a baseline for the efficient use of patients’ health records. Still, the lack of normalized operational guidelines and competent staff compromises the efficacy of these archives and has led to gaps in communication in CB-PHC settings [45]. Such gaps do not merely impair the quality of continuing care at PHC levels but also compromise treatment in higher-tier hospitals by weakening referrals. Holistic reforms, which can enhance the management of health information in CB-PHC settings, improve the coordination and integration of various healthcare institutions, facilitate referrals, and replace fee-for-service with hybrid payment approaches, must be introduced [39,42].

Consistent with existing reviews [14,15,16,17], our review identifies that staff shortages and poor training in CB-PHC settings are significant organizational barriers. Many recent studies reveal that the workforce is vital to patient management in CB-PHC settings [13,45]. The number and level of training of community health workers are significant predicators of service utilization, quality of health supervision, and the proper use of medical equipment [51,54]. Although painstaking efforts have been made to address these issues by Chinese authorities, such as intensified university education for medical students, the cosmetic and unsystematic on-the-job training provided for active health workers considerably impairs human resources in CB-PHC settings [13]. In addition, poor working conditions, low pay, and lack of social security facilitate the brain drain observed in CB-PHC settings [13,53]. Stimulus strategies, which can both scale up the workforce and upskill active health workers for a “stay-in-and-do-well” working landscape, should be a top priority on the health policy agenda.

Our review also highlights the inadequacy of high-quality medical equipment in the CB-PHC sector. As a result of the long-standing hospital-centered service delivery model, large public hospitals in China receive ample resources to purchase state-of-the-art equipment. Community health organizations, in contrast, have no such resources; the consequent uneven distribution of equipment means that CB-PHC is considered inferior and is avoided. Given the generalized perception of Chinese citizens that large public hospitals are more dependable, the second-rate equipment used in CB-PHC settings appears to be a key facilitator for the robustness and persistence of “too difficult to see a doctor” in urban China. If there are no orchestrated schemes aimed to advance the equipment in CB-PHC settings, the demands for PHC among residents will have to be replenished by higher-tier hospitals, and the envisioned functions of CB-PHC in health promotion will be consequently bottlenecked.

With a few exceptions [41,50,51], most included studies did not employ a specific conceptual framework to make clear causal inferences about the barriers. Instead, they merely provided snapshots of the determinants and features of the sample using a cross-sectional approach. Consequently, we do not have solid evidence to confirm the causes of the barriers through this review. Longitudinal research and qualitative case studies with specific conceptual frameworks are warranted to ascertain the causalities.

More than 70% of the included studies were conducted in East China, indicating a geographic partiality on this topic. Constrained by poorer economic conditions, non-eastern regions of China often lack sufficient funding to develop CB-PHC infrastructure. They also suffer from the absence of qualified community health practitioners. As a result, these regions are more likely to encounter obstacles in care delivery. Scholars need to direct their attention to the relevant issues in the underdeveloped regions of China and offer policy recommendations for their CB-PHC development.

In addition, as we found, more of the included studies focused on the issues of people-centered care between 2012 and 2016, whereas more on organization and management problems emerged from 2017 to 2021. This shift in research areas of interest indicates that scholars have started to pay more attention to organizational and structural issues in CB-PHC settings, which may have a considerable impact on CB-PHC development in urban China and bring about systematic changes in community health. Nevertheless, people-centered care is still worth researching as it is a core practice for empowering patients throughout the course of care [56]. Ideally, holistic studies covering both people-centered care and organizational issues are warranted.

Furthermore, access-related barriers require further exploration. Brainstorming to overcome barriers arising from people-centered care and organization and management is essential to inform policy change. Still, reflections on barriers to access should not be marginalized. After all, access to the right care represents the most basic value of CB-PHC: health for all.

## 5. Conclusions

Having synthesized the selected evidence, our review identified six significant barriers to care delivery: lack of comprehensive health insurance schemes, lack of public awareness, superficial care relationships, gaps in communication, staff shortages and poor training, and second-rate equipment. These barriers have clearly jeopardized health promotion within urban communities. Given the complicated and far-reaching nature of these barriers, piecemeal reforms are no longer adequate. To fulfill the envisioned functions of CB-PHC, targeted policy interventions are needed. Therefore, we propose several recommendations for future policy reforms. First, the government needs to take action to further integrate the current social health insurance schemes by adopting more (re)centralized strategies. Second, it is necessary to innovate and multiply incentives for community health practitioners and the general public to improve the service utilization rate. Third, the “Medical Alliance” (*yilianti*) framework has great potential to bridge an effective communication channel between CB-PHC organizations and other healthcare institutions and is, therefore, worth more input from the government. Last, but not least, there is a need to institutionalize professional training for practitioners and enact targeted human resources management regulations in CB-PHC settings.

Following a rigorous, transparent, and objective process, our systematic mapping review provides a panorama of the barriers to CB-PHC delivery in urban China and their impact on community-based health care. The breadth of evidence it captures makes our findings valuable to further systematic reviews, empirical research, and policy analysis. Still, two caveats resulting from our review should be noted. First, we dismissed the subdomain “availability of effective care” in the PHCPI framework, which might make our search results less complete. Also, most of the included studies focused on East China, which may hamper the generalization of our review. Future syntheses should address these concerns.

## Figures and Tables

**Table 1:** Services provided by CB-PHC and non-CB-PHC sectors in urban China.

Key PHC Service Domains	CB-PHC Organizations	Non-CB-PHC Organizations		
	Community Health Service Centers and Stations	Sub-District Health Service Centers	Clinics	Outpatient Departments
Prevention	*√*	*√*	*X*	*√*
Treatment	*√*	*√*	*√*	*√*
Rehabilitation	*√*	*√*	*X*	*√*
Palliative care	*√*	*X*	*X*	*X*
Health education	*√*	*√*	*X*	*X*

Abbreviation: CB-PHC—community-based primary health care. *√*—available; *X*—not available.

**Table 2:** Grouping and characteristics of the included studies.

Study	Methods	Sample	Sample Size	Research Sites
Theme one: Access				
Yin et al. (2019)	Cross-sectional	Patients (diabetes)	1691	Multi-provinces
Sun et al. (2019)	Cross-sectional	Residents (aged ≥18)	915	Sichuan
Li … & Mao. (2019)	Cross-sectional	Migrants (hypertension, aged >18)	1046	Shenzhen
Li … & Hu. (2019)	Cross-sectional	Patients (hypertension, aged ≥18)	867	Shenzhen
Zhong et al. (2018)	Cross-sectional	Patients (aged ≥18)	1461	Guangdong
Liu, D. et al. (2017)	Cross-sectional	Residents (aged ≥18)	2247	Chengdu
Li, W. et al. (2017)	Cross-sectional	Patients (aged ≥18)	6887	Shenzhen
Gan et al. (2016)	Cross-sectional	Patients (aged 18-90)	7761	Shenzhen
Chung et al. (2016)	Cross-sectional	Patients (aged ≥18)	3360	Guangdong *
Zeng et al. (2015)	Cross-sectional	Migrants (aged ≥18)	736	Guangzhou
Shi et al. (2015)	Cross-sectional	Patients (hypertension, diabetes, aged ≥50)	560	Guangdong
Chung et al. (2013)	Cross-sectional	Patients (aged ≥18)	3356	Guangdong *
Theme two: People-centered care				
Zhang, W. et al. (2020)	Cross-sectional	Residents (aged ≥60)	2754	Nationwide
Zhang, T. et al. (2020)	Cross-sectional	Patients (aged ≥18)	624	Hangzhou
Zhang, L. et al. (2020)	Cross-sectional	Patients (aged ≥18)	515	Changchun
Yue et al. (2020)	Interview	Patients (chronic disease) & nurses	26	Beijing
Gu et al. (2020)	Cross-sectional	Residents (aged ≥60)	480	Nanjing
Pu et al. (2019)	Cross-sectional	Patients (PTB, aged ≥15)	638	Guizhou
Huang et al. (2019)	Cross-sectional	Residents (aged ≥15)	2919	Shanghai
Su et al. (2017)	Cross-sectional	Residents (hypertension)	1,092,031	Nationwide
Qian et al. (2017)	Interview	Medical staff & patients	50	Hangzhou
Liu, C. et al. (2017)	Cross-sectional	Patients (aged ≥18)	700	Beijing
Li, H. et al. (2017)	Longitudinal	Patients (hypertension, aged ≥60)	880	Shanghai
Li, H. et al. (2016)	Cross-sectional	Patients (hypertension, aged 18-80)	782	Wuhan, Nanjing
Gu et al. (2016)	Cohort	Patients (heart disease, aged ≥55)	329	Beijing
Zhong et al. (2015)	Cross-sectional	Community inhabitants	9067	Anhui
Li … Yang et al. (2015)	Cross-sectional	Patients (hypertension, aged ≥18)	696	Shanghai, Shenzhen
Li … Lao et al. (2015)	Cohort	Patients (hypertension, aged ≥18)	3196	Shanghai, Shenzhen
Kuang et al. (2015)	Cross-sectional	Patients (aged ≥18)	1645	Guangdong
Du et al. (2015)	Cross-sectional	Patients	864	Guangdong
Yang et al. (2014)	Cross-sectional	Residents (NCD, aged ≥18)	51,501	Guangdong
Wang et al. (2014)	Cross-sectional	Residents	162,464	Guangdong
McCollum et al. (2014)	Cross-sectional & interview	Patients (aged ≥18) & health directors	231 + 8	Fuzhou
Chen et al. (2014)	Cross-sectional	Patients (hypertension, aged ≥35)	3191	Chengdu
Wang et al. (2013)	Cross-sectional	Patients (aged ≥18)	1440	Guangdong *
Shao et al. (2013)	Cross-sectional	Residents (aged ≥15)	6592	Beijing
Theme three: Organization and management				
Liu et al. (2021)	Longitudinal	Doctors	8968	Shanghai
Huang et al. (2021)	Longitudinal	Residents (aged ≥18)	4749	Shanghai
Zhang et al. (2020)	Cross-sectional & interview	Residents & health workers	989 + 32	Guizhou, Chongqing
Yao et al. (2020)	Cross-sectional	Patients (diabetes) & care providers	2610	Shandong
Xia et al. (2020)	Cross-sectional	Physicians & nurses	2719	Nationwide
Duan et al. (2020)	Cross-sectional	Patients (diabetes, aged ≥18)	1972	Yueqing
Zhu et al. (2019)	Cross-sectional	Patients (diabetes, common illness, aged ≥18)	816	Hangzhou
Zhao et al. (2019)	Interview	General practitioners	32	Beijing
Zhan et al. (2019)	Cross-sectional	Outpatient prescribers	150	Sichuan
Wang et al. (2019)	Cross-sectional	Patients (diabetes, aged ≥18)	1598	Shandong, Jiangsu
Searle et al. (2019)	Interview	Medical leaders	17	Shenzhen
Liang et al. (2019)	Cross-sectional & interview	Health workers & leaders	198 + 70	Guizhou, Chongqing
Huang et al. (2019)	Longitudinal	Patients (NCD, aged ≥18)	4749	Shanghai
Chen et al. (2019)	Cross-sectional	General practitioners & nurses	172	Shanghai
Zhu et al. (2018)	Interview	Managers & physicians	15	Wuhan
Mao et al. (2018)	Interview	Childcare providers	22	Hunan
Li, W. et al. (2018)	Cross-sectional	Patients (aged ≥18)	1159	Wuhan
Li, L. et al. (2018)	Cross-sectional	Patients (aged 18-89)	698	Guangzhou
Zhang et al. (2017)	Cross-sectional	Primary care workers	791	Multi-provinces
Wu et al. (2017)	Cross-sectional & interview	Residents	1248 + 19	Hangzhou
Wong et al. (2017)	Cross-sectional	Clinicians & primary care practitioners	3738	Nationwide
Wei et al. (2017)	Cross-sectional	Primary care users	2924	Multi-cities
Ong et al. (2017)	Cross-sectional	Primary care practitioners	3580	Nationwide
Wu et al. (2016)	Cross-sectional	Patients (aged ≥18)	3848	Shenzhen
Li, J. et al. (2016)	Cross-sectional	Patients (aged ≥15)	1918	Jilin
Chapman et al. (2016)	Interview	Doctors	23	Shenzhen
Wei et al. (2015)	Interview	Managerial & professional staff	60	Guangdong *
Jing et al. (2015)	Longitudinal	Residents (aged ≥18)	1200	Shanghai
Wang et al. (2014)	Interview	Primary family caregivers	23	Not specific
Li et al. (2014)	Cross-sectional	Patients (aged ≥18)	787	Shenzhen
Wong et al. (2012)	Cross-sectional	Patients (hypertension)	1830	Guangdong *

* Research sites in the literature were the Pearl River Delta Region, geographically comprising the nine cities within Guangdong Province. Abbreviations: PTB—pulmonary tuberculosis; NCD—noncommunicable disease.

**Table 3:** Barriers to CB-PHC delivery and their impacts on community-based health care in urban China identified by this review.

Barriers to CB-PHC Delivery	Impacts of the Barrier on Community-Based Health Care
PHCPI subdomain: Access	
Lack of comprehensive health insurance schemes	○ Lowering the likelihood of being routinely treated in CB-PHC facilities among the uninsured
	○ Leading to unsupervised and unsafe medication use by the uninsured
	○ Making CB-PHC benefits inaccessible to migrants without local health insurance
	○ Resulting in delayed access to CB-PHC facilities among migrants covered by non-local health insurance
	○ Leading to substandard procedures, outcomes, and perceived quality of CB-PHC among migrants
	○ Decreasing the willingness of migrants to join local health insurance schemes
Lack of public awareness	○ Resulting in poor awareness of CB-PHC and little willingness by residents to use the services
	○ Decreasing the trust and satisfaction in CB-PHC of residents with low SES
	○ Contributing to the overuse of high-tier hospitals and waste of health resources in CB-PHC settings
PHCPI subdomain: People-centered care	
Superficial care relationships	○ Lowering users’ trust in providers
	○ Generating inefficient communication between users and providers
	○ Lowering the ability of users to perceive the benefit of modern medicines
	○ Impeding information exchange during the treatment process
	○ Lowering the transparency of treatment
	○ Decreasing users’ assessment of service quality
	○ Diminishing the sense of reciprocity and rapport between users and providers
	○ Diminishing the trust of providers in users’ cooperation and self-administration
	○ Making the responses to users’ concerns inadequate and ineffective
Gaps in communication	○ Resulting in delayed feedback on the effects of medication
	○ Making recovery and continued treatment inefficient
	○ Making regular follow-up examinations unlikely and, if provided, inefficient
	○ Compromising the referral system
	○ Contributing to medical errors and complications
	○ Discouraging care coordination and integration
	○ Escalating episodic medical costs and rates of hospitalization
PHCPI subdomain: Organization and management	
Staff shortages and poor training	○ Lowering the utilization rate of CB-PHC
	○ Increasing the workloads of active health workers
	○ Contributing to care provider burnout
	○ Leading to disparities in the service quality of health workers
	○ Reducing the likelihood that residents would choose to receive CB-PHC
	○ Resulting in brain drain in CB-PHC settings
	○ Compromising the professional development of health workers
	○ Lowering the ability of health workers to practice effective care
	○ Impeding the application and expansion of digitalization of medical services
	○ Undermining the modernization of the national healthcare system
Second-rate equipment	○ Making it difficult to keep up-to-date medical records
	○ Lowering functionality in medical data acquisition, referral arrangements, and information exchange and interoperability
	○ Hampering innovative care practices
	○ Contributing to the inadequacy of the professional education of health workers
	○ Decreasing residents’ desire to join health-promoting activities
	○ Contributing to the growing over-reliance on specialist care in higher-tier hospitals and underuse of general services provided by community health organizations

## Data Availability

No new data were created or analyzed in this study. Data sharing is not applicable to this article.

## Supplementary Materials

The following supporting information can be downloaded at: https://www.mdpi.com/article/10.3390/ijerph191912701/s1, Table S1: Quality assessment of the included studies using qualitative methods [27]. Table S2: Quality assessment of the included studies using quantitative methods [26]. Table S3: A priori review protocol [34].
