# Supplementary material for: Barriers to Community-Based Primary Health Care Delivery in Urban China: A Systematic Mapping Review
Source: Int J Environ Res Public Health. 2022 Oct 4;19(19):12701. doi: 10.3390/ijerph191912701 (PMC9566097; doi:10.3390/ijerph191912701)
Supplement: Supplementary file 1 [file ijerph-19-12701-s001.zip › ijerph-1864544-supplementary.pdf]

Table S1: Quality assessment of the included studies using qualitative methods [27].

Study	Critical Appraisal Skills Program Checklist (Yes/Good; Can't tell/Moderate; No/Poor)									
	Was there a clear statement of the aims of the research?	Is a qualitative methodology appropriate?	Was the research design appropriate to address the aims of the research?	Was the recruitment strategy appropriate to the aims of the research?	Was the data collected in a way that addressed the research issue?	Has the relationship between researcher and participants been adequately considered?	Have ethical issues been taken into consideration?	Was the data analysis sufficiently rigorous?	Is there a clear statement of findings?	How valuable is the research?
Yue et al. (2020)	Yes	Yes	Yes	Yes	Yes	Yes	Yes	Yes	Yes	Good
Zhang et al. (2020)	Yes	Can't tell	Yes	Yes	Yes	Yes	Yes	Yes	Yes	Good
Zhao et al. (2019)	Yes	Can't tell	Yes	Yes	Yes	No	Yes	Can't tell	Yes	Moderate
Liang et al. (2019)	Yes	Yes	Yes	Yes	Yes	Yes	Yes	Yes	Yes	Good
Searle et al. (2019)	Yes	Yes	Yes	Yes	Yes	Can't tell	Yes	Yes	Yes	Good
Zhu et al. (2018)	Yes	Yes	Yes	Yes	Yes	Yes	Yes	Yes	Yes	Good
Mao et al. (2018)	Yes	Yes	Yes	Yes	Yes	Can't tell	Yes	Yes	Yes	Good
Wu et al. (2017)	Yes	Can't tell	Yes	Yes	Can't tell	Can't tell	Yes	Yes	Yes	Moderate
Qian et al. (2017)	Yes	Yes	Yes	Can't tell	Yes	Can't tell	Yes	Yes	Yes	Good
Chapman et al. (2016)	Yes	Yes	Yes	Yes	Yes	Can't tell	Yes	Yes	Yes	Good
Wei et al. (2015)	Yes	Can't tell	Yes	Can't tell	Yes	Yes	Yes	Yes	Yes	Moderate
Wang et al. (2014	Yes	Yes	Yes	Yes	Yes	Yes	Yes	Yes	Yes	Good
McCollum et al. (2014)	Yes	Yes	Yes	Can't tell	Can't tell	No	Yes	Yes	Yes	Good

Table S2: Quality assessment of the included studies using quantitative methods [26].

Study	Downs and Black Score (out of 28*)	Quality level**	Study	Downs and Black Score (out of 28*)	Quality level**
Liu et al. (2021)	21	Good	Li, H. et al. (2017)	21	Good
Huang et al. (2021)	22	Good	Zhang et al. (2017)	26	Excellence
Zhang et al. (2020)	18	Fair	Wu et al. (2017)	27	Excellence
Yao et al. (2020)	21	Good	Wong et al. (2017)	21	Good
Xia et al. (2020)	22	Good	Wei et al. (2017)	22	Good
Duan et al. (2020)	17	Fair	Ong et al. (2017)	19	Fair
Zhang, W. et al. (2020)	19	Fair	Li, J. et al. (2016)	18	Fair
Zhang, T. et al. (2020)	22	Good	Li, H. et al. (2016)	19	Fair
Zhang, L. et al. (2020)	21	Good	Gu et al. (2016)	22	Good
Gu et al. (2020)	26	Excellence	Gan et al. (2016)	23	Good
Yin et al. (2019)	23	Good	Chung et al. (2016)	21	Good
Sun et al. (2019)	19	Fair	Wu et al. (2016)	24	Good
Li … & Mao. (2019)	21	Good	Jing et al. (2015)	20	Good
Li … & Hu. (2019)	22	Good	Zhong et al. (2015)	24	Good
Pu et al. (2019)	21	Good	Li … Yang et al. (2015)	22	Good
Huang et al. (2019)	20	Good	Li … Lao et al. (2015)	16	Fair
Zhu et al. (2019)	19	Fair	Kuang et al. (2015)	15	Fair
Zhan et al. (2019)	21	Good	Du et al. (2015)	19	Fair
Wang et al. (2019)	18	Fair	Zeng et al. (2015)	22	Good
Liang et al. (2019)	19	Fair	Shi et al. (2015)	24	Good
Huang et al. (2019)	17	Fair	Wang et al. (2014)	21	Good
Chen et al. (2019)	22	Good	McCollum et al. (2014)	22	Good
Li, W. et al. (2018)	23	Good	Chen et al. (2014)	24	Good
Li, L. et al. (2018)	26	Excellence	Li et al. (2014)	20	Good
Zhong et al. (2018)	22	Good	Wang et al. (2013)	23	Good
Liu, D. et al. (2017)	21	Good	Shao et al. (2013)	21	Good
Li, W. et al. (2017)	20	Good	Chung et al. (2013)	22	Good
Su et al. (2017)	19	Fair	Wong et al. (2012)	19	Fair
Liu, C. et al. (2017)	18	Fair			

* In the present version of the checklist we modified the scoring of item 27 that refers to the power of the study. Instead of rating according to an available range of study powers, we rated whether the study or not performed power calculation. Accordingly, the maximum score for item 27 was instead of 5 and thus the highest possible score for the checklist was 28 (instead of 32). ** The score ranges were given corresponding quality levels as previously reported [28]: excellent (26-28); good (20-25); fair (15-19); and poor (≤14).

Table S3: A priori review protocol [34].

Section	Item	Checklist item
Title		
• Identification	1a	Barriers to Community-Based Primary Health Care Delivery in Urban China: A Systematic Mapping Review
• Update	1b	Not applicable
Registration	2	Not applicable
Authors		
• Contact	3a	Bo Li<br>bo-li.li@connect.polyu.hk<br>Department of Applied Social Sciences, The Hong Kong Polytechnic University, Hung Hom, Kowloon, Hong Kong<br>Juan Chen<br>juan.chen@polyu.edu.hk<br>Department of Applied Social Sciences, The Hong Kong Polytechnic University, Hung Hom, Kowloon, Hong Kong
• Contributions	3b	Conceptualization, B.L. and J.C.; methodology, B.L. and J.C.; software, B.L.; validation, B.L. and J.C.; formal analysis, B.L. and J.C.; investigation, B.L. and J.C.; resources, B.L. and J.C.; data curation, B.L. and J.C.; writing—original draft preparation, B.L.; writing—review and editing, J.C.; visualization, B.L.; supervision, J.C.; project administration, J.C.; funding acquisition, J.C.
Amendments	4	Not applicable
Support		
• Sources	5a	Electronic databases
• Sponsor/funder	5b	The Hong Kong Polytechnic University Mental Health Research Centre (Project ID: P0040455)
• Role of sponsor/funder	5c	Proofreading
INTRODUCTION		
Rationale	6	Existing reviews on China's CB-PHC have focused primarily on quality issues, while there has been little synthesis of the barriers to care delivery. This paper aims to fill this gap through a systematic mapping review.
Objective/research questions	7	• Objective: to synthesize selected evidence on the barriers to CB-PHC delivery in urban China and their impact on community-based health care.<br>• Research questions:<br>o What are the barriers to CB-PHC delivery in urban China?<br>o What are the impacts of the barriers on community-based health care?
METHODS		
Eligibility criteria	8	• Inclusion criteria: peer-reviewed English and Chinese academic journal articles published between 2012 and 2021 (both qualitative and quantitative research).<br>• Exclusion criteria: 1) studies using non-empirical data; 2) studies focusing on regions outside mainland China; 3) studies that do not focus on the urban population; 4) studies using data collected before or in 2009; 5) grey literature (e.g., policy documents, consultancy reports, research notes, commentaries, editorials, letters, correspondence); 6) review articles; 7) randomized trials; 8) pilot studies; and 9) study protocols.

Information sources	9	Electronic databases (CINAHL, MEDLINE, Scopus, Web of Science, and CNKI) and hand searches of references cited in existing reviews.
Search strategy	10	• Terms and queries (Boolean operators and Medical Subject Headings used to instruct our searches and optimize the breadth of searching results):<br>o On the English databases: 'China' OR 'Chinese' AND 'community' OR 'community-based' AND 'primary care' OR 'primary healthcare' OR 'primary health care' AND 'service delivery' OR 'care delivery' OR 'delivery of services' OR 'delivery of care' OR 'access' OR 'accessibility' OR 'first contact' OR 'coordination' OR 'comprehensiveness' OR 'continuity' OR 'safety' OR 'facility management' OR 'team-based care' OR 'supervision' OR 'population health management' OR 'information systems' OR 'monitoring' OR 'quality management.'<br>o On the Chinese database: '中国' OR '我国' AND '社区' OR '社区为本' OR '社区导向' AND '初级保健' OR '初级卫生保健' OR '基层医疗' OR '基层保健' OR '社区医疗' AND '服务提供' OR '服务传递' OR '服务实施.'<br>• The terms and queries were informed by the PHCPI framework.
Study records		
• Data management	11a	EndNote X9 and Microsoft Excel
• Selection process	11b	• 499 articles identified, 113 duplicates removed, titles and abstracts of the 386 remaining articles screened, 300 articles excluded (due to either irrelevance or exclusion criteria), the full text of the 80 articles retrieved and assessed, 13 articles removed after assessment, 67 articles included in the final sample.<br>• The selection process was conducted by the authors independently.
• Data collection process	11c	A predetermined form was used to extract the data. The data collection process was completed by the authors independently.
Data items	12	• Outcome variables: barriers to CB-PHC delivery, impacts of the barriers on community-based health care.<br>• Characteristics of the articles: years of publication, research methods, study sample, regions under study, and others (specified in Table 1).
Outcomes and prioritization	13	See the main text.
Risk and bias in individual studies	14	The Downs and Black and the CASP checklists were used to assess the quality of eligible papers.
Data		
Syntheses	15	Thematic narrative syntheses were performed; close, open, and axial coding were applied; ATLAS.ti (version 9) software was used in data coding and analysis.
Meta-bias	16	Not applicable
Confidence in cumulative evidence	17	Not applicable
